# Induction of killing of *Mycobacterium avium* subsp. *hominissuis* in macrophages by cytokine stimulated innate-like lymphoid cells is negatively affected by the pathogen

**DOI:** 10.1007/s10123-023-00326-4

**Published:** 2023-01-20

**Authors:** Jay Bickel, Luiz E Bermudez

**Affiliations:** 1 Department of Biomedical Science, College of Veterinary Medicine, Oregon State University, Corvallis, OR USA; 2 Department of Microbiology, College of Science, Oregon State University, Corvallis, OR USA

**Keywords:** *Mycobacterium avium* subsp. *hominissuis*, Innate-like lymphoid cells, Macrophage, MAH, Interferon gamma, IL-4, Il-12, Il-17, IL-22, IL-33, Mouse, Spleen

## Abstract

*Mycobacterium avium* subsp. *hominissuis* (MAH) is a common environmental bacterium that causes infection in immunocompromised patients such as those with HIV/AIDS, or patients with chronic lung disease such as cystic fibrosis. There are many strains of MAH with varying levels of virulence. Infection with MAH strains 100 and 104 has been associated with different immune responses in mice and outcome of the disease. While MAH 100 infection tends to be cleared from mice, MAH 104 is virulent and grows in host tissue. What is currently unknown are the mechanisms related to this difference in host defense and virulence. Our hypothesis is that differences in circulating innate lymphocytes response are associated with increased protection from infection. Innate lymphoid cells (ILC) are lymphoid cells with an important role in regulation of innate immune systems. ILCs can be categorized into three subpopulations ILC1, ILC2, and ILC3 based on their cytokine production and regulatory transcription factors. Investigation was carried out on how macrophage anti-MAH response change depending on activation by primary mouse lymphocytes activated with IL-12, IL-33, and IL-23, triggering differentiation into ILC-like subpopulations. Our results do not affirm the role of any one ILC subpopulation in macrophage anti-*M. avium* ability. Our findings instead support the conclusion that MAH infection of macrophages suppresses the stimulatory function of ILCs.

## Introduction

*Mycobacterium avium* subs. *hominissuis* (MAH) is an opportunistic pathogen that exists ubiquitously in the environment (Griffith et al. 2007; Horseburgh 1999). It is associated with disease in immunocompromised patients and individuals with chronic lung disease such as cystic fibrosis, bronchiectasis, and chronic obstructive pulmonary disease (Griffith et al. 2007; Horseburgh 1999; Prevots and Marras 2015). Present in environmental soil and water, as well as household sources such as shower heads, dust, and pools, MAH is acquired via inhalation or ingestion (Griffith et al. 2007; Prevots and Marras 2015). Infection is difficult to treat, requiring lengthy courses of multiple antibiotics, and therefore presents a significant public health issue (Griffith et al. 2007; Horseburgh 1999).

Following ingestion or inhalation, and subsequent crossing of intestinal or respiratory mucosa, MAH infects macrophages. From here, the bacterium is capable of altering the host cell response in order to increase their chance of survival; for example, one strategy employed by MAH is to prevent acidification of macrophage phagosomes (Sturgill-Koszycki et al. 1994). Bacteria can then replicate intracellularly before spreading locally or disseminating throughout the body (Bermudez et al. 2015; Saunders et al. 2002).

Host immune response to MAH infections are not fully understood. Many strains of MAH have varying levels of virulence. Infection with MAH strains 100 and 104, for example, have been associated with different immune responses in mice (Saunders et al. 2002). While MAH 100 infection tends to be cleared from mice, MAH 104 has a greater virulence and grows in host tissue (Saunders et al. 2002). What is currently unknown are the mechanisms which would explain this difference in virulence.

The aim of this work was to examine whether these differences in virulence are partially due to the roles of innate-like lymphoid cells in the host immune response to MAH. Innate lymphoid cells (ILCs) are a newly described cell population that localize in different tissues and have varied roles in tissue homeostasis but are been shown to be increasingly relevant players in the complex field of the immune system (Eberl et al. 2015; Gasteiger et al. 2015). ILCs can be thought of as the bridge that gaps innate and adaptive immunity, as they respond to cellular stimulation with the release of cytokines to drive immune responses (Eberl et al. 2015; Gasteiger et al. 2015). Specifically, ILCs are part of the complex and flexible immunologic network important in inflammatory conditions and innate immune response. They are found in abundance within mucosal surfaces, highlighting their potential importance as a line of defense against infectious disease (Gasteiger et al. 2015; Ardain et al. 2019). Furthermore, ILCs can be categorized into five different subsets, natural killer cells (NK), ILC1, ILC2, ILC3, and lymphoid tissue inducer (LTi) subpopulations based on their differing functions*.* All ILC subpopulations can be encountered in the lungs and blood of healthy humans and are very likely stimulated upon contact with infectious agents (Diefenbach et al. 2020). MAH bacteremia also have been shown to play a role in modulating the presence of these ILC subpopulations during and after *Mycobacterium tuberculosis* infection, affirming the significance of these cells during infection (Diefenbach et al. 2020; Mazzurana et al. 2018).

The three different subpopulations of ILCs are defined by the first-order cytokines which differentiate them and then the second-order cytokines which are secreted in response to challenge infection (Mazzurana et al. 2018). ILC1 cells are activated by interleukin (IL)-12 leading to the production of interferon gamma (IFN-γ), and have similar function to type 1 helper T-cells playing an important and multi-faceted role in responding to intracellular pathogen (Mazzurana et al. 2018; Spits et al. 2016; Mortha and Burrows 2018). Additionally, ILC1 functions resemble natural killer (NK) cells in many aspects, such as being capable of stimulating infected macrophages to fight the infection (Eberl et al. 2015; Mortha and Burrows 2018). IFN-γ released by ILC1 acts on macrophages to control mycobacterium infections by activating their antimicrobial properties (Sousa and Rastogi 1992).

ILC2 cells are activated by IL-33 and secrete IL-4 having a similar role to type 2 helper T-cells. These cells are encountered in the lungs at higher levels than other ILC subpopulations before infections arise, and play a role in surveillance of airway epithelium and maintenance of the epithelium when damaged (Mazzurana et al. 2018). Raised levels of IL-4 are detected in MAH infections; however, their role in T-cell regulation are not fully defined (Ricardo-Gonzales et al. 2018). Evidence in *M. tuberculosis* infections suggests they have an early effect in T-cell regulation, macrophage activation, and inflammation that determine whether the infection becomes latent or progressive (Rook et al. 2004).

ILC3 cells are stimulated by IL-23 and produce IL-17 similar to their counterpart TH17 cells (Mortha and Burrows 2018). ILC3 has been shown to have an early protective role in mycobacterium infections, which leads to rapid accumulation of ILC3 in lung tissues which coincides with accumulation of alveolar macrophages (Mortha and Burrows 2018; Michel et al. 2012; Marashian et al. 2015). Overall, this ILC subpopulation has the most diverse roles of the ILC subpopulations and these roles are shaped by environmental conditions in the body (Marashian et al. 2015).

Investigation was carried out on how macrophages responded to activation by primary mouse lymphocytes belonging to the three innate lymphoid cell-like subpopulations. It is important to note that in this experimental model, mouse lymphocytes were harvested from the spleen. This was done in part due to the relative ease of harvesting circulatory lymphocytes rather than mucosal ones. While the lungs can directly contact respiratory pathogens, the spleen is a lymphatic organ that is in contact with the circulatory system to stimulate or suppress immune responses (Michel et al. 2012). ILCs replenish and self-maintain locally leading to distinct microenvironments of these cells in each organ that been shown to cause phenotypic and functional differences of lymphocytes (Mazzurana et al. 2018). In both the lung and spleen environments, lymphocytes are regulated by the surrounding cells. For example, alveolar macrophages inhibit NK activity while spleen macrophages do not, but are able to further prime NK cytotoxicity and proliferation (Michel et al. 2012). Additionally, in the spleen, lymphocytes can be exposed to phagocytic and endothelial cells (Michel et al. 2012), whereas in the lungs, epithelial cells secrete cytokines upon contact with pathogens for cell differentiation; in the circulation, differentiation relies on cytokines produced by phagocytic cells, circulating lymphocytes, and endothelial cells (Michel et al. 2012). Consideration of these microenvironment differences and their relevance to results will be further discussed in this report.

By infecting primary mouse spleen lymphocytes to obtain supernatant representing differentiated ILC-like subpopulations, we intended to determine whether ILC-derived supernatant would have any role in the stimulation of macrophages, with the ability to suppress MAH infection. Specifically, it was hypothesized that at least one of the three ILC populations play a significant role in the immune response to MAH and activate macrophage’s ability to inhibit MAH infection.

## Materials and methods

### Bacterial strains

Two strains of *Mycobacterium avium* (MAH) 100 and 104 were used. MAH strain 104, originally isolated from the blood of an AIDS patient, causes disseminated and pulmonary infection in mice, while MAH 100, also isolated from the blood of AIDS patient, is attenuated in mice (Saunders et al. 2002; Jeffrey et al. 2017). Both strains were grown on Middlebrook 7H10 agar supplemented with 10% w/v oleic acid-albumin-dextrose-catalase (OADC; Hardy Diagnostics; Santa Maria, Ca) and used between 7 and 14 days of growth and 1–5 passages in vitro. All strains were grown at 37°C. Inoculums for all assays were prepared in Hanks Balanced Salt Solution (HBSS, Cellgro, Manassas, VA) and syringe passaged for dispersion of clumping in the suspension before establishing the inoculum using a spectrophotometer. Appropriate suspension was then prepared to a multiplicity of infection (MOI) of 1. *Escherichia coli* HB101 (K12) strain and *Staphylococcus epidermidis* (skin isolate) were cultured on Muller-Hinton agar at 37°C. At the time of the experiment, bacteria were suspended in HBSS and the inoculum prepared as described above.

### Host cells

Peritoneal murine macrophage RAW 264.7 cell line obtained from the American Type Culture Collection (ATCC; Manassas, VA) was cultured in Roswell Park Memorial Institute Medium 1640 (RPMI-1640; Corning) supplemented with 10% fetal bovine serum (FBSl, Gemini Bio-products). Macrophage numbers for infection assays were determined with a hemocytometer and seeded at 60% confluency in 48-well tissue culture plates. Monolayers were then infected 24 h later when confluency reached approximately 80%. Because RAW 264.7 cells continue to replicate after infection, 100 μL of fresh media was added every 48 h to support cell growth.

### Supernatant preparation

Spleens (approved by the Oregon State University IACUC) were harvested from C57BL/6 mice to tease the tissue in RPMI using two sterile 22-gauge needles until there were no signs of organized tissue. Media containing cells were then transferred into tissue culture plate and incubated at 37°C for 45 min. Once macrophages adhered to the plastic, lymphocytes and media were removed by aspiration and then the plates were washed with fresh RPMI-1640 medium. CD3+ splenic cells were selected using specific antibody as previously reported (Mohagheghpour et al. 1997). Adherent macrophages were then washed with HBSS twice and medium was replenished with fresh RPMI-1640 supplemented with 10% fetal bovine serum (FBS, Gemini Bio-products). ILC-like subpopulations (10^6^ cells/mL) 1, 2, and 3 were differentiated with the addition 10ng/mL of either recombinant mouse IL-12, Il-33, or IL-23 (Genzyme), respectively, to tissue culture plate wells containing either lymphocytes or lymphocytes and macrophages. All cells were given 24 h to rest before being infected with MAH 104 at MOI of 1. The lymphocytes (1×10^6^ cells/mL) were then exposed to cytokines triggering differentiation into ILC-like subpopulations. Controls included wells with non-stimulated cells as well as wells with heat-killed MAH 104. Supernatants were then collected directly at 4, 24, and 48 h post-infection, and syringe filtered (2-μm filter) before storage at −20°C for use in future assays.

### Survival assays

Monolayers of RAW 264.7 cells at 60% confluency (5×10^5^ cells) were seeded and given 24 h to adhere to wells before been infected with either MAH 100 or MAH 104 strain of MAH for 1 h at a MOI of 1. Inoculums were standardized between experiments using optical density measurements and confirmed through dilution, plating, and incubation at 37°C for 7–10 days in order to determine the number of viable colonies. HBSS was then used to rinse monolayers and remove the remaining extracellular bacteria. Different treatments were then added to monolayers infected with either MAH 100 or MAH 104. *E. coli* and *S. epidermidis* were used as non-virulent controls. Monolayers were infected with 1×10^5^ bacteria.

#### Supernatant treatment

In some assays, supernatants were added onto pairs of wells, each for them infected with MAH 100, MAH 104, *E. coli*, or *S. epidermidis*. Briefly, supernatants were unfrozen, brought up to 37°C, and vortex agitated before addition to wells. Control wells were incubated with supernatant from heat-killed bacteria and additional control wells were incubated with RPMI+ 10% FBS and absent of supernatant.

In alternative assays, 1μL of 10ng/mL of recombinant IL-4, IL-17, or IFN-γ was added to monolayers infected with MAH 100 or MAH 104. Control wells were incubated in RPMI-1640 +10% FBS with no added cytokines. Each assay was performed in triplicate.

#### Supernatant and cytokine treatment

In some assays, 100 μL of ILC1 differentiated supernatant and 1μL IFN-γ (10 ng/mL) were added to duplicate wells of a monolayer infected MAH 104. Controls included supernatant with heat-killed bacteria and wells incubated in RPMI-1640 supplemented with 10% FBS only, with no IFN-γ.

In some assays, infected macrophages (5×10^5^) were incubated with supernatants, in the presence of rabbit anti-mouse IFN-γ antibody (10 mg/mL).

For all of the above described assays, at days 2 and 4 post-infection, the monolayers were lysed in 0.1% Triton-X100 (Sigma Aldrich, St. Louis, MO) for 10 min followed by pipetting the lysate in and out of well. Contents of wells were then serially diluted and plated onto Middlebrook 7H10 agar to quantify the number of viable bacteria present in infection. Plates were incubated at 37°C for 7–10 days until visible colonies formed for counting.

### Statistical analysis

All results are representative of either duplicate or triplicate replicates as indicated. Significance and standard deviation were calculated for all the assays. GraphPad Prism and Excel were used for all statistical analysis. Comparison between treatment groups and controls was determined using a two-tailed *t*-test and confirmed with a 2-way ANOVA. Significant values had *p*<0.05. All graphs were created using GraphPad Prism and tables and figures were created using Microsoft Suite.

## Results

### ICL-like lymphocytes’ ability to stimulate macrophages

**Fig. 1:**
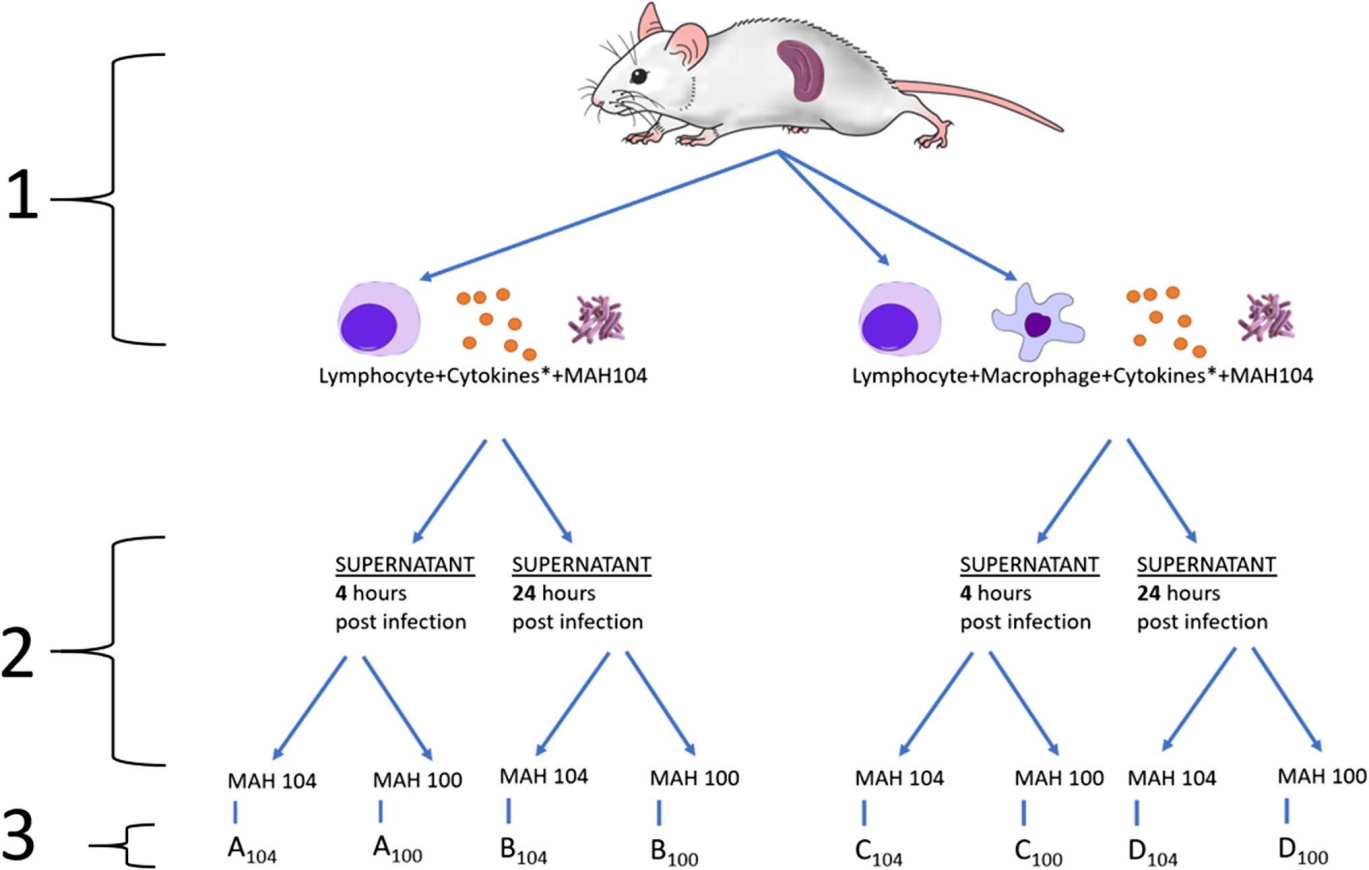
Experimental design. (1) Primary lymphocytes or macrophages harvested from a mouse spleen, infected with MAH 104 and activated with *IL-12, IL-23, or IL-33. (2) Supernatant from infected mouse cells added to MAH 100 and 104 RAW 264.7 cell infections. (3) Coded result condition groups for reference in results and discussion

The schematic representation of the used mice protocol is shown in Fig. 1. To determine whether the supernatant of the three ILC-like subpopulations induced the ability to stimulate macrophages to inhibit or kill intracellular *M. avium*, CFUs were determined from a survival assay. RAW 264.7 macrophage monolayers were infected with either MAH 100 or MAH 104 and then exposed to previously collected supernatants (4 h or 24 h) for the duration of the experiment.

**Fig. 2:**
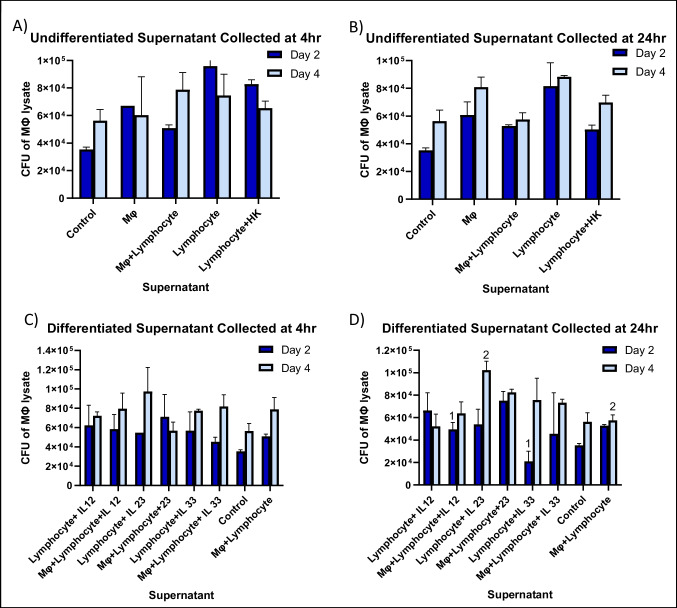
To determine whether macrophages infected with MAH 100 respond to stimulation with supernatant of splenic lymphocytes exposed to IL-12, IL-23, or IL-33, a survival assay was performed. RAW cells (5 × 10^5^ cells) were infected with MAH 100 (1×10^6^) bacteria and differentiated ILC subpopulation supernatants (from 10^6^ cells/mL). Control wells contained fresh RPMI+10%FBS media and no supernatant. Supernatant controls included undifferentiated cells and cells infected with heat-killed (HK) bacteria. Macrophage monolayers were lysed at 2 days and 4 days to determine intracellular bacteria at each time point as CFUs. Statistically significant results are indicated with corresponding numbers (*p*<0.05) using two-way ANOVA and two-tailed *t*-test. #1: *p* < 0.05 compared to control at day 2. #2: *p* < 0.05 comparing Mo + lymphocyte treatment with lymphocyte + IL-23. Day 0 macrophages (infection had **A** 2.2±0.3 × 10^4^; **B** 2.5 ± 0.2 × 10^4^; **C** 3.1 ± 0.2 × 10^4^; **D** 2.0 ± 0.2 × 10^4^)

Monolayers infected with MAH 100 showed no significant differences between control groups (supernatants of undifferentiated lymphocytes) and any of the ILC-like groups from the C_100_ conditions (Fig. 2). This trend remained similar for ILC-like groups from D_100_ conditions with the exception of IL-23 differentiated lymphocytes showing significantly higher CFUs than macrophage and lymphocyte control supernatants (Fig. 2).

**Fig. 3:**
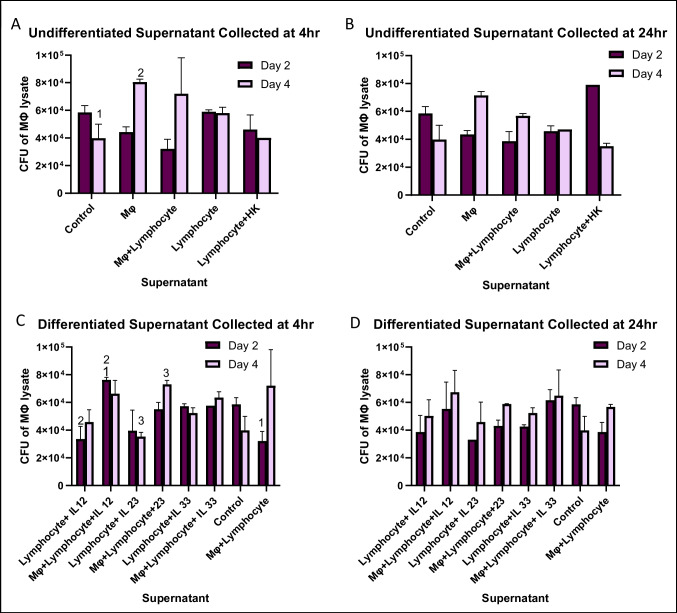
To determine whether macrophages infected with MAH 104 respond to stimulation with supernatant of splenic lymphocytes treated with IL-12, IL-23, or IL-33, a survival assay was performed. RAW cells (5 × 10^5^ cells) were infected with MAH 104 (1×10^6^) and differentiated ILC subpopulation supernatants (from 10^6^ cells/mL). Control wells contained fresh RPMI+10% FBS media and no supernatant. Supernatant controls included undifferentiated cells and cells infected with heat-killed (HK) bacteria. Macrophage monolayers were lysed at 2 days and 4 days to determine intracellular bacteria at each time point as CFUs. Statistically significant results are indicated with corresponding numbers (*p*<0.05) using the 2-way ANOVA and two-tailed *t*-test. #1: *p* < 0.05 compared to day 0 macrophages (infection had **A** 4.8 ± 0.3 ×10^4^). #2: *p* < 0.05 comparing day 4 Mo with day 2 Mo. Macrophage infection at day 0, **B** 4.8 ± 0.3 × 10^4^; macrophage infection at day 0, **C** 4.7 ± 0.2 × 10^4^; #1: *p* < 0.05 comparing Mo + lymphocyte _ IL-12 at day 2 with control, and with Mo + lymphocyte at day 2. #2: *p* < 0.05 comparing lymphocyte + IL-12 with Mo + lymphocyte + IL-12 at day 2. #3: *p* < 0.05 comparing lymphocyte + IL-23 with Mo + lymphocyte + IL-23 at day 4. Macrophage at day 0, **D** 4.4 ± 0.3 × 10^4^

When monolayers were infected with MAH 104, no significant differences were observed between control groups (supernatants of undifferentiated lymphocytes) and any of the ILC-like differentiated groups from the A_104_, B_104_, C_104_, or the D_104_ conditions (Fig. 3). However, for each ILC-like subpopulations in A_104_, there were significantly lower CFUs than the corresponding ILC-like subpopulation activated from C_104_ wells. For example, A_104_ wells containing supernatant derived from lymphocytes activated with IL-12 showed a 56% reduction in CFUs compared to C_104_ wells containing supernatant derived from both lymphocytes and macrophages activated with IL-12. This trend was also seen between B_104_ and D_104_ ILC subpopulations; however, they were not statistically significant.

In general, the results show that there is not a great effect on macrophage activation when comparing different ILC-like subpopulations for either MAH 100 or MAH 104 infections. Instead, the results demonstrated significant decreases of MAH 104 CFUs in ILC-like subpopulations derived from lymphocytes only, rather than lymphocyte and macrophage combination. The results were unexpected and raised the question of whether the expected ILC-produced cytokines were present in the supernatant and if they could have an impact with direct stimulation rather than indirect introduction through activation into ILC-like subpopulations. As the next step, it was to assess the question by establishing a baseline of direct addition of ILC-like produced cytokines to macrophages. As controls, RAW 264.7 macrophages were infected with either *E. coli* K12 or *S. epidermidis*. As shown in Table 1, supernatants of ILC subpopulations 1 and 3 induced macrophages to kill intracellular bacteria, confirming the killing-induced capability of the ILC supernatants.

**Table 1:** Ability of the supernatant of activated ILCs to stimulate macrophage bactericidal phenotype

Infection	Treatment	CFU/mL of lysate	
	Supernatant	4 h	48 h
*E. coli*		6.3 ± 0.6 × 10^4^	3.7 ± 0.5 × 10^2^
	ILC1		5.1 ± 0.4 × 10^1^*
	ILC2		2.9 ± 0.7 × 10^2^
	ILC3		4.4 ± 0.3 × 10^1^*
*S. epidermidis*		2.4 + 0.4 × 10^4^	7.9 ± 0.4 × 10^1^
	ILC1		2.5 ± 0.3 × 10^1^*
	ILC2		8.7 ± 0.4 × 10^1^
	ILC3		2.3 ± 0.4 × 10^1^*

*p* < 0.05 compared with untreated control. The results represent the mean ± SD of 3 individual experiments (ANOVA). Macrophage monolayers with 5×10^5^ cells. Supernatants collected from 10^6^ cells/mL

* *p* < 0.05

### Do stimulated macrophage suppress MAH growth?

RAW 264.7 cells were infected with either MAH 104 or MAH 100, and then stimulated with recombinant IL-4, IL-17, and IFN-γ to determine if purified cytokines were associated with indirect activation of ILC-like subpopulations.

**Fig. 4:**
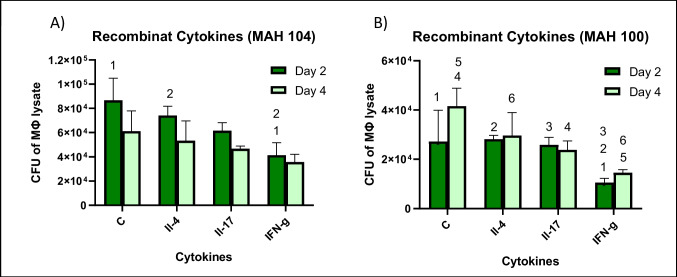
To determine whether macrophages infected with MAH 100 or MAH 104 respond to stimulation with cytokines IL-4, IL-17, or IFN-γ, a survival assay was performed. RAW cells (5 × 10^5^ cells) were infected with MAH 100 or MAH 104 (MOI of 1) and cytokines were added to wells. Control wells contained fresh RPMI+10% FBS media and no cytokines. Macrophage monolayers were lysed at 2 days and 4 days to determine intracellular bacterial load at each time point as CFUs. Statistically significant results are indicated with corresponding numbers (*p*<0.05) using two-tailed *t*-test and 2-way ANOVA. Day 0 macrophages (infection had **A** 4.3 ± 0.3 × 10^4^). #1: *p*< 0.05 comparing control with IFN-g treatment at day 2. #2: *p* < 0.05 comparing IFN-g treatment with IL treatment at day 2. Macrophage CFU at time 0, **B** 1.1 ± 0.4 × 10^4^. #1: *p* < 0.05 comparing control and IFN-g at day 2; #2: *p* < 0.05 comparing IL-4 treatment with IFN-g treatment at day 2; #3: *p* < 0.05 comparing IL-17 treatment with IFN-g treatment at day 2; #4: *p* < 0.05 comparing control with IL-17 treatment at day 4; #5: *p* < 0.05 comparing control with IFN-g at day 4; #6: *p* < 0.05 comparing IL-4 treatment with IFN-g treatment at day 4

Beginning 24 h after infection, visually IFN-γ-treated wells showed change in morphology as they formed long spindles and became highly vacuolized. This was neither seen in other wells of the recombinant cytokine experiment nor the original previous experiment containing IFN-γ ILC-like producing subpopulations. IFN-γ-treated macrophages infected with MAH 104 showed a statistically significant decrease in CFUs compared to control wells. On day 2, IFN-γ-treated wells had a 52% decrease from day 2 control cells, while at day 4, IFN-γ-treated wells had a 42% decrease in bacterial load (Fig. 4, panel A). This activation of macrophages was even more pronounced in MAH 100 infection where IFN-γ-treated wells had statistically lower CFU counts compared to control and all other recombinant cytokine-treated wells (Fig. 4, panel B).

The results of this assay did not align with the results of the initial assay using supernatant treatments, since a clear difference in ability to reduce CFUs in this case was seen between different treatments. Because the cytokines or other products secreted by the lymphocytes could be diverse and not successfully identified, we opted by an alternative approach. One possible reason for this discrepancy was that the supernatant would contain product(s) that are neutralizing the ILC-like produced cytokines. In order to investigate if this was potentially occurring, a new experiment was designed that combined supernatant with cytokines.

### Supernatants of ICL-like lymphocytes block macrophage response to stimulation

**Fig. 5:**
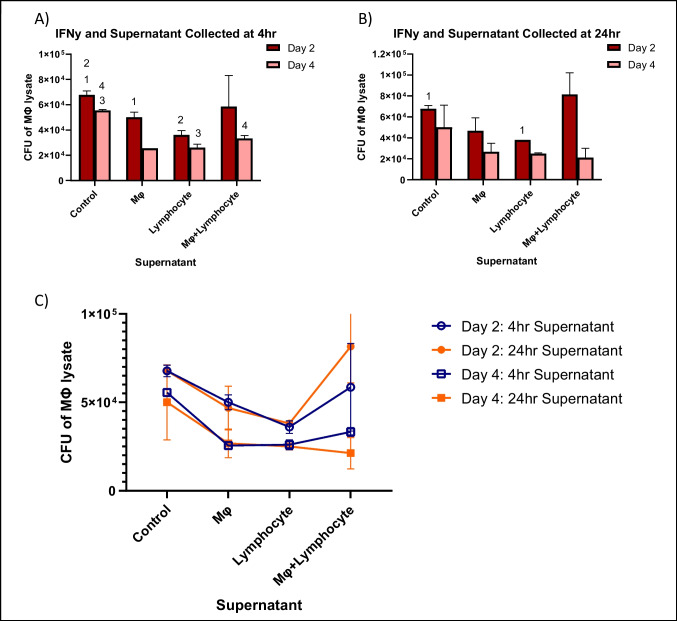
To determine whether macrophages infected with MAH 104 respond to stimulation with supernatant of splenic lymphocytes treated with IL-12, as well as direct IFN-γ stimulation, a survival assay was performed. RAW cells (5×10^5^ cells) were infected with MAH 104 (MOI of 1) and differentiated ILC-like subpopulation supernatants (from 10^6^ cells/mL). Control wells contained fresh RPMI+10% FBS media and no supernatant. Macrophage monolayers were lysed at 2 days and 4 days to determine the number of viable intracellular bacteria at each time point as CFUs. Statistically significant results are indicated with corresponding numbers (*p*<0.05) using two-tailed *t*-test and 2-way ANOVA. Macrophage at day 0, CFU: 4.5 ± 0.3 × 10^4^. In panel A: #1 *p*< 0.05 comparing control with Mo at day 2; #2 *p*< 0.05 comparing control with lymphocyte at day 2; #3 *p* < 0.05 comparing control with lymphocyte at day 4; #4 *p* < 0.05 comparing control with Mo + lymphocyte at day 4

Following infection of macrophages with MAH 104, ILC1 differentiated supernatant collected at 4 and 24 h post-infection and IFN-γ were added to wells. Resulting CFUs showed statistically significant decrease of survival in all treatment groups containing supernatant derived from primary macrophages that was collected 4 h after infection compared to controls (Fig. 5, panel A). In contrast, treatment with supernatant collected 24 h post-infection showed little significant ability of macrophages to kill the bacterium on either day 2 or 4 of the assay compared to controls (Fig. 5, panel B). The only statistically significant supernatant condition collected at 24 h was day 2 lymphocyte supernatant which was 44% less than the day 2 control. In the presence of IFN-γ, exposure to supernatant collected 24 h after infection has less effect on macrophage ability to decrease bacteria in infection than supernatant collected after 4 h (Fig. 5, panel C).

To determine if the presence of anti-IFN-γ antibody would have any effect on the ability of macrophages to control MAH 104 growth, antibody was added to infected as well as treated macrophage monolayers in addition to supernatants. The results showed that the treatment of macrophages with specific antibody for IFN-γ demonstrates that IFN-γ has only small influence of macrophage activation (Table 2).

**Table 2:** Effect of anti-IFN-γ antibody on the ability of supernatant of ILC1-like subpopulation of lymphocytes to stimulate anti-MAH acidity of macrophages

Experimental group	CFU/mL of lysate	Decrease compared to control
Macrophage + MAH 104 (control)	3.6 ± 0.3 × 10^5^	
Macrophage + MAH 104 + supernatant 4 days	2.9 ± 0.4 × 10^5^	20%
Macrophage + MAH104 + supernatant 4 days + anti-IFN-γ	3.2 ± 0.5 × 10^5^	11%
Macrophage + MAH 104 + supernatant 4 days + IFN-γ	9.4 ± 0.5 × 10^4^	67.6%*

RAW 264.7 mouse macrophages (5×10^5^ cells). Infected MOI of 1 for 1 h

Results represent the mean +/− of three individual experiments

*p < 0.05 compared to macrophage control (ANOVA)

## Discussion

MAH is an important ubiquitous environmental pathogen that causes disease in immunocompromised patients (Griffith et al. 2007; Horseburgh 1999; Prevots and Marras 2015). After ingestion or inhalation, MAH bacteria pass through mucosal layers to infect host macrophages where they survive and replicate in intracellular vacuoles (Sturgill-Koszycki et al. 1994). While it is understood that MAH bacteria are capable of altering macrophage phagosomes to survive intracellularly, their methods of immune evasion are still largely unknown (Sano et al. 1998; Wagner et al. 2002). The aim of this study was to examine the potential role of innate lymphocyte cells (ILC) on the host defense in MAH infections. ILCs are unspecialized cells that exist in both the mucosa and circulation, where they respond to cytokine signals to differentiate and secrete cytokines to influence surrounding cells such as macrophages in an innate manner of host defense (Mazzurana et al. 2018). Our findings suggest that in the presence of macrophages and lymphocytes, MAH 104 bacterium triggers macrophages to be less responsive to ILC1-like produced IFN-γ.

Using a system with macrophage cultures in vitro and macrophage and lymphocytes ex vivo, it was observed that applying activated lymphocyte supernatant treatment on peritoneal macrophages infected with MAH 100 or MAH 104, in general, led to no difference on macrophage killing abilities. MAH 100 and MAH 104 behave differently in mice with MAH 104 being the more virulent of the two (Saunders et al. 2002; Jeffrey et al. 2017). It was hypothesized that a specific ILC subpopulation may be responsible for this difference, but the obtained results indicated no impact from an ILC subpopulation on either 100 or 104 infection. In fact, what is responsible for the differences in mouse infection outcome does not seem to be related to the macrophage function. It is still possible that the stimulation of airway ICL, instead of circulating ones, may provide different results. In case the mucosal ICLs show some aspect of immunologic memory, they might have a role in the defense against environmental bacteria (Zeis et al. 2020; Starkey et al. 2019).

These findings suggest that the response to *M. avium* may be different than *Mycobacterium tuberculosis*. Previous work with *M. tuberculosis* showed that ILC3 subpopulations are able to mediate early protection against the bacterium (Ardain et al. 2019). However, while MAH and *M. tuberculosis* have many similarities, they trigger different responses in macrophages upon activation with cytokines (Carvalho de Sousa and Rastogi 1992). Previous work has shown that pre-infection treatment of macrophages with IFN-γ does not confer any anti-MAH response, while the same treatment confers significant anti-*M. tuberculosis* properties to macrophages which may explain this difference (Carvalho de Sousa and Rastogi 1992). Additionally, it was found in this initial assay that in general, MAH 104-infected lymphocyte supernatants had statistically lower CFUs than lymphocyte and macrophage supernatants. Many of the functions of lymphocytes are to assist macrophages fight against infection, and these interactions only occur when the cells are in close proximity to macrophages. In contrast, MAH 100 infections showed no significant difference at 4 h and limited significance at 24 h. This data alternatively suggests that MAH 104 may interfere with normal macrophage and lymphocyte interactions when both are present.

In our study, although did not observe a significant role for ICLs, other possibilities can explain our results. The role of antibodies is one possibility. Antigen present ICLs can induce IgA response to bacteria in the mucosa, as has been demonstrated by other group (Melo-Gonzales et al. 2019). That would be one step further than our study explored, and should be addressed in the future.

In order to further explore the potential suppressive effects of the supernatant, a second survival assay looking at direct macrophage activation of MAH 100- and MAH 104-infected macrophages with recombinant cytokines was performed. This was carried out using the second-order cytokines expected to be produced and released in the supernatant for each ILC subpopulation in order to establish the anticipated effect of the supernatant on macrophage infection. Results showed a significant decrease in CFUs for IFN-γ-treated MAH 104 and an even greater decrease in CFUs for MAH 100 infections. IFN-γ is known to upregulate anti-MAH 104 activity in macrophages and not be produced in significant quantities in MAH 100 infections (Saunders et al. 2002; Sano et al. 1998). However, even though MAH 100 infections do not typically produce IFN-γ, it is not unexpected that it had a strong impact on macrophage ability to reduce infection because IFN-γ supports macrophage ability to clear intracellular pathogens.

In order to look at the impact of IFN-γ stimulation directly in conjunction with ILC1 (IFN-γ-producing) supernatant on MAH 104 infections, a survival assay was performed where both were added post-infection. Wells with supernatant collected 4 h post-infection show a significant decrease in macrophage killing ability, whereas wells with supernatant collected 24 h post-infection show significantly less ability for macrophages to limit MAH 104 infection. These results suggest that when exposed to MAH 104, the effect of IFN-γ is suppressed by the actions of the supernatant. The presence of this effect is most apparent in supernatant collected 24 h post-infection suggesting that this is a response that occurs over time as host cells and bacteria interact.

As demonstrated in the assay of macrophage infection in the presence of recombinant cytokines, as well as literature, IFN-γ clearly plays a role in antimycobacterial activity (Sano et al. 1998; Appelberg and Orme 1993). However, this effect was neither apparent in the original assay of macrophage infection in the presence of supernatant nor the final assay of supernatant in conjunction with direct IFN-γ stimulation. Literature analysis shows that IFN-γ treatment of MAH macrophages confers less macrophage bacteriostatic abilities than macrophages infected with *M. tuberculosis* (Saunders et al. 2002). In the scope of this study, this discrepancy of IFN-γ impact on MAH infection may be explained by the presence of some product(s) produced upon activation of ILC subpopulation in the presence of macrophages and lymphocytes that block the effect of IFN-γ.

There are many possibilities regarding what is responsible for the decreased impact of supernatant on IFN-γ. It has been shown in literature that *Mycobacterium avium* infections cause an increased output of IL-10 that is implicated in MAH pathogenesis (Denis and Ghadirian 1993; Bermudez and Champsi 1993). When treated with IL-10 neutralizing antibodies, mouse resistance to infections is conferred (Denis and Ghadirian 1993; Bermudez and Champsi 1993). Additionally, similar results are seen with mice treated with TGF-beta and TGF-beta neutralizing infusions, where TGF-beta leads to increased MAH growth that is counteracted when neutralized (Bermudez 1993). While IL-10 synthesis and secretion begin shortly after initiation of infection, TGF-beta levels are first detected at day 3 (Sano et al. 1998). This is one potential difference between the difference in supernatant collected 2 days post-infections versus 4 days that leads to blockage of IFN-γ effect and decreased anti-MAH capabilities.

Further work needs to be done to address the limitations in the scope of this study. Firstly, as introduced earlier, spleen- and lung-derived lymphocytes interact with different cells to activate and differentiate. While these differences in activation are known, it has not been determined whether once activated they have the same outputs. Spleen-derived lymphocytes were used in this research because of their relative ease to harvest compared to lung-derived lymphocytes, but future work may benefit from directly comparing the results of this work when done with spleen- versus lung-derived lymphocytes. Secondly, future work should measure the levels of different ILC-produced cytokines in supernatant as well as resulting supernatant from macrophage infection in the presence of recombinant cytokines and supernatant to determine whether cytokines such as IFN-γ are present in effective levels or not present at all.

MAH infections do not cause illness in the average individual, but for AIDS patients and individuals with chronic lung disease, MAH infections can become serious and expensive, requiring long treatments with multiple drugs. The role of ILCs in these infections is still not fully known. This study has shown that MAH infection can interfere with ILC1 activity and ultimately diminish the effects of IFN-γ. In the future, better understanding of ILC roles in MAH infections and immune responses will lead to better treatment options.

## Data Availability

All the raw data and specific materials used in the reported research are available upon request to Dr. Luiz E Bermudez.

## References

[CR1] Appelberg R, Orme LM (1993). Effector mechanisms involved in cytokine-mediated bacteriostasis of Mycobacterium avium infections in murine macrophages. Immunology.

[CR2] Ardain A, Domingo-Gonzalez R, Khader S (2019). Group 3 innate lymphoid cells mediate early protective immunity against tuberculosis. Nature.

[CR3] Bermudez L, Champsi J (1993). Infection with *Mycobacterium avium* Induces Production of Interlukin-10 (IL-10), and administration of Anti-IL-10 Antibody is Associated with Enhanced Resistance to Infection in Mice. Infect Immun.

[CR4] Bermudez LE (1993). Production of transforming growth factor beta-1 by *Mycobacterium avium* infected macrophages is associated with unresponsiveness to interferon gamma. J Immunol.

[CR5] Bermudez LE, Danelishvili L, Babrak L (2015). Tuan Pham. Evidence for genes associated with the ability of *Mycobacterium avium* subsp. *hominissuis* to escape apoptotic macrophages. Front Cell Infect Microbiol.

[CR6] Carvalho de Sousa J, Rastogi N (1992). Comparative ability of human monocytes and macrophages to control the intracellular growth of *Mycobacterium avium* and *Mycobacterium tuberculosis*: effect of interferon-gamma and indomethacin. FEMS Microbiol Immunol.

[CR7] Denis M, Ghadirian E (1993). IL-10 neutralization augments mouse resistance to systemic *Mycobacterium avium* infections. J Immunol.

[CR8] Diefenbach A, Gnafakis S, Shomrat O (2020). Innate lymphoid cell-epithelial cell modulates sustain intestinal homeostasis. Immunity.

[CR9] Eberl G, Colonna M, Santo J, McKenzie A (2015). Innate lymphoid cells: a new paradigm in immunology. Science.

[CR10] Gasteiger G, Fan X, Dikiy S, Lee S, Rudensky A (2015). Tissue residency of innate lymphoid cells in lymphoid and nonlymphoid organs. Science.

[CR11] Griffith DE, Aksamit T, Brown-Elliott BA, Catanzaro A, Daley C, Gordin F, Holland SM, Horsburgh R, Huitt G, Iademarco MF, Iseman M, Olivier K, Ruoss S, Reyn CFV, Wallace RJ, Winthrop K (2007). An official ATS/IDSA statement: diagnosis, treatment, and prevention of nontuberculous mycobacterial diseases. Am J Respir Crit Care Med.

[CR12] Horseburgh R (1999). The pathophysiology of disseminated *Mycobacterium avium* complex disease in AIDS. J Infect Dis.

[CR13] Jeffrey B, Rose S, Gilbert K, Lewis M, Bermudez LE (2017). Comparative genomic analysis regarding pathogenesis of four *Mycobacterium avium* subsp. *hominissuis* clinical isolates. J Med Microbiol.

[CR14] Marashian S, Mortaz E, Jamaati H, Alavi-Moghaddam M, Kiani A, Abedini A, Garssen J, Adcock I, Velayati A (2015). Role of innate lymphoid cell in lung disease. Iran J Allergy Asthma Immunol.

[CR15] Mazzurana L, Rao A, Acker A, Mjosberg J (2018) The roles for innate lymphoid cells in the human immune system. Semin Immunopathol 40(4): 407–417 10.1007/s00281-018-0688-7 PMC6060849 29948108

[CR16] Melo-Gonzales F, Kammoun H, Dutton EE, Papadopoulou M, Bradford BM, Tanes C, Reid FF, Swann JR, Bittinger K, Mabbott NA, Vallence BA, Willinger T, Withers DR, Hepworth MR (2019) Antigen-presenting ICL3 regulates T cell -dependent IgA responses to colonic mucosal bacteria. J Exp Med :20180871 10.1084/jem.20180871 PMC6446868 30814299

[CR17] Michel T, Poli A, Mauffray M, Theresine M, Brons N, Hentges F, Zimmer J (2012). Mouse lung and spleen natural killer cells have phenotypic and functional differences, in part influenced by macrophages. PLoS One.

[CR18] Mohagheghpour N, Gammon D, Vollenhoven A, Hornig Y, Bermudez LE, Young LS (1997). Mycobacterium avium reduces expression of costimulatory/adhesion molecules in human monocytes. Cell Immunol.

[CR19] Mortha A, Burrows K (2018). Cytokine networks between innate lymphoid cells and myeloid cells. Front Immunol.

[CR20] Prevots DR, Marras TK (2015). Epidemiology of human pulmonary infection with nontuberculous mycobacteria: a review. Clin Chest Med.

[CR21] Ricardo-Gonzales RR, Van Dyken SJ, Schneider C, Lee J, Nussbaum JC, Liang HE, Vaka D, Eckalbar WL, Molofsky AB, Erie DJ, Locksley RM (2018). Tissue signals imprint ILC2 identity with anticipatory function. Nat Immunol.

[CR22] Rook G, Hernandez-Pando R, Dheda K, Seah G (2004). IL-4 in tuberculosis: implications for vaccine design. Trends Immunol.

[CR23] Sano C, Sato K, Shimizu T, Kajitani H, Kawauchi H, Tomioka H (1998). The modulating effects of proinflammatory cytokines interferon-gamma (IFNγ) and tumor necrosis factor-alpha (TNF-a) and immunoregulating cytokines IL-10 and transforming growth factor (TGF-beta) on anti-microbial activity of murine peritoneal macrophages against *Mycobacterium avium-intracellulare* complex. J Leukoc Biol.

[CR24] Saunders D, Dane A, Briscoe H, Britton W (2002). Characterization of immune responses during infection with *Mycobacterium avium* strains 100, 101 and the recently sequenced 104. Immunol Cell Biol.

[CR25] Sousa J, Rastogi N (1992). Comparative ability of human monocytes and macrophages to control the intracellular growth of *Mycobacterium avium* and *Mycobacterium tuberculosis:* effect of interferon-gamma and indomethacin. FEMS Microbiol Immunol.

[CR26] Spits H, Bernink J, Lanier L (2016). NK cells and type 1 innate lymphoid cells: partners in host defense. Nat Immunol.

[CR27] Starkey MR, McKenzie ANJ, Belz GT, Hansbro PM (2019). Pulmonary group 2 lymphoid cells: surprises and challenges. Mucosal Immunol.

[CR28] Sturgill-Koszycki S, Schlesinger PH, Chakraborty P, Haddix PL, Collins HL, Fok AK, Allen RD, Gluck SL, Heuser J, Russell DG (1994). Lack of acidification in Mycobacterium phagosomes produced by exclusion of the vesicular proton-ATPase. Science.

[CR29] Wagner D, Sangari FJ, Kim S, Petrofsky M, Bermudez LE (2002). Mycobacterium avium infection of macrophages results in progressive suppression interleukin-12 production *in vitro* and *in vivo*. J Leukoc Biol.

[CR30] Zeis P, Lian M, Herman J, Hernandez D, Gentec R, Elias S, Symowski C, Knooper K, Peltokanas N, Friederich C (2020). In situ maturation and tissue adaptation of type 2 innate lymphoid cell progenitors. Immunity.

